# Editorial: Biofilms in aquatic environments and new strategies for microbial biofilm control

**DOI:** 10.3389/fmicb.2026.1960548

**Published:** 2026-08-21

**Authors:** Jorge T. Antunes, Inês Bezerra Gomes, Lorenzo Proia

**Affiliations:** 1 Universidade Católica Portuguesa, CBQF—Centro de Biotecnologia e Química Fina—Laboratório Associado, Escola Superior de Biotecnologia, Porto, Portugal; 2 LEPABE—Laboratory for Process Engineering, Environment, Biotechnology and Energy, University of Porto, Porto, Portugal; 3 ALiCE—Associate Laboratory in Chemical Engineering, Faculty of Engineering, University of Porto, Porto, Portugal; 4 BETA Technological Centre–University of Vic–Central University of Catalonia (BETA–UVIC–UCC), Barcelona, Spain

**Keywords:** antimicrobial resistance, antimicrobials, aquatic environment, biofilm control, biofilms, Aquatic biofilms

Aquatic biofilms are ubiquitous in both natural and engineered environments. As highly complex and diverse microbial communities, they play fundamental roles in ecosystem functioning and nutrient cycling. However, biofilms can also negatively affect water quality, compromise the performance and stability of infrastructure and industrial equipment, and act as reservoirs of pathogenic microorganisms. Furthermore, biofilms are closely associated with the emergence and dissemination of antimicrobial resistance. Therefore, advancing our understanding of biofilm characteristics, dynamics and functions in aquatic environments is essential to assess their impacts on environmental sustainability, public health and industrial systems, as well as to develop innovative and effective strategies to mitigate biofilm-related problems.

**Figure 1:**
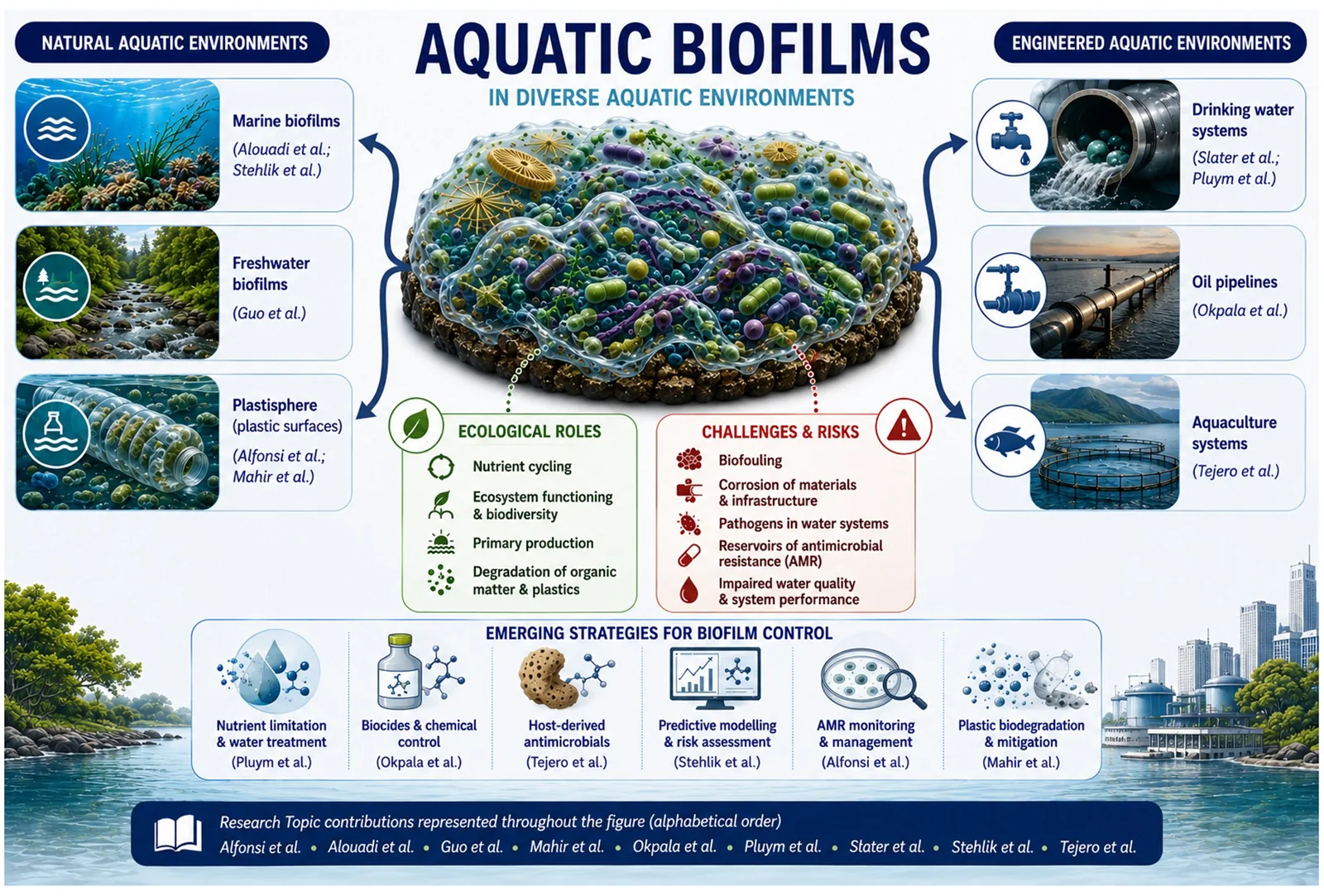
Overview of the studies published in the Research Topic “Biofilms in aquatic environments and new strategies for microbial biofilm control”.

The articles included in this Research Topic present recent advances in our understanding of aquatic biofilms across a range of environments, including marine, freshwater and drinking water systems, as well as their colonization of engineered structures and plastic surfaces. Using diverse experimental approaches, these studies provide new insights into biofilm formation, structure, function, and ecological dynamics, while also exploring control strategies where these microbial communities represent an engineering or public health challenge. Collectively, these studies highlight the relevance of aquatic biofilms to key environmental, industrial, and public health challenges, with important implications for water quality management, biofouling and corrosion control, and mitigation of antimicrobial resistance in aquatic ecosystems (Figure 1).

Some works published in this Research Topic focused on biofilms formed in engineered aquatic environments, such as drinking water systems and submersed crude oil-transporting pipelines, providing significant insights on their ecology and diversity (Slater et al.) and on the impact of different strategies for microbial and biofilm mitigation (Pluym et al. and Okpala et al.).

Slater et al. investigated whether the composition of drinking water biofilms is mainly determined by the planktonic community of the supplying water, or by more constant environmental conditions within the distribution system itself. Using three full-scale pipe-loop facilities fed by distinct source waters (surface reservoir, river, and groundwater) and disinfection regimes, the authors tracked bacterial and fungal biofilm and planktonic communities over 12 months. Although the three planktonic microbiomes remained clearly distinct throughout the study, the corresponding biofilm communities converged toward a similar bacterial and fungal composition within 3 months of growth, differing mainly in relative abundance rather than in membership. This convergence points to shared selective pressures, most likely pipe material and hydraulic regime, which were held constant across the three systems, as the main drivers of biofilm community assembly, rather than the inoculating bulk water, an insight relevant to biofilm management in drinking water distribution networks.

Also addressing challenges in drinking water distribution systems, Pluym et al. investigated the effectiveness of ultrafiltration (UF) and nanofiltration (NF) in reducing total organic carbon (TOC) to improve the biological stability of distributed drinking water without, or with minimal chlorination. Using a pilot-scale drinking water distribution system, the authors compared the impact of UF and NF treatment on microbial dynamics in both bulk water and biofilms. NF achieved substantially greater TOC removal than UF. Although both treatments resulted in similar increases in planktonic cell concentrations during distribution, they promoted distinct microbial communities in bulk water, while biofilm communities remained largely stable despite differences in nutrient availability. Furthermore, invasion assays with *Aeromonas media, Pseudomonas putida*, and *Serratia fonticola* demonstrated enhanced decay of these introduced microorganisms in UF- and NF-treated water. Collectively, these findings highlight the importance of reducing biodegradable organic matter to improve drinking water biostability and increase resistance to microbial invasion in non- or minimally chlorinated distribution systems. These findings demonstrate the value of preventive approaches that enhance the biological stability of drinking water by limiting nutrient availability. Nevertheless, preventing microbial proliferation alone may not be sufficient once mature biofilms have developed, emphasizing the need for effective biofilm control strategies.

The work of Okpala et al. evaluates chemical strategies for controlling sulfate-reducing microorganism (SRM) biofilms using biomass collected during routine cleaning of a crude oil transmission pipeline. Biofilms were established under dynamic conditions in a flow cell system and exposed to two chemical treatments: sodium nitroprusside (SNP) and alkyl dimethyl benzyl ammonium chloride (ADBAC). ADBAC is a quaternary ammonium compound widely used as a disinfectant in industrial settings, including cooling towers, heat exchangers, and oilfield systems (Xie et al., 2019). SNP, a vasodilator commonly used in clinical practice, has also attracted interest as an antibiofilm agent in applications such as membrane fouling control and the inhibition of sulfate-reducing bacteria in oil-related systems (Fida et al., 2018). The study demonstrated that established biofilms were considerably more tolerant to both compounds than planktonic cultures. While low concentrations effectively inhibited planktonic cells, biofilms required substantially higher doses, with SNP producing only transient inhibition even at the highest concentration tested, whereas ADBAC completely suppressed sulfidogenesis only at 250–500 ppm. Furthermore, 16S rRNA gene sequencing revealed that active biofilms were dominated by *Desulfobulbus* together with either *Desulfomicrobium* or *Pseudomonas*, with these taxa showing differential persistence following biocide exposure. Collectively, these findings highlight the intrinsic resilience of established SRM biofilms and demonstrate that conventional biocide dosing strategies may provide only temporary control, emphasizing the need for more effective and sustainable approaches to mitigate biofilm-associated microbiologically influenced corrosion.

Other contributions to this Research Topic examined biofilms colonizing natural and anthropogenic surfaces across aquatic environments, addressing their diversity, ecological drivers, ecological functions, and role in the dissemination of antimicrobial resistance (Alfonsi et al.; Mahir et al.; Guo et al.).

Alfonsi et al. investigated the plastisphere, the biofilm community that develops on plastic debris, as a potential reservoir and vector of antimicrobial resistance (AMR) in a river of central Italy. By deploying caged polyethylene and polypropylene fragments alongside water sampling, the authors recovered a substantial proportion of multidrug-resistant, cefotaxime-resistant Enterobacteriaceae from both plastic-associated biofilms and the water column, including carbapenem-resistant strains producing carbapenemases: KPC (*Klebsiella pneumoniae* carbapenemase) and NDM (New Delhi metallo-β-lactamase). Shotgun metagenomics of the plastisphere further revealed a resistome enriched in genes conferring resistance to clinically relevant antibiotic classes, together with mobile genetic elements, chiefly plasmids and Class 1 integrons, capable of mediating horizontal gene transfer. Gene cassettes carried by integrons from plastic-associated bacteria were more heterogeneous than those from the water column, reinforcing the plastisphere's potential to concentrate and diversify resistance determinants and highlighting riverine plastic pollution as a route through which clinically relevant AMR may re-enter human populations.

Building on the focus on the plastisphere, Mahir et al. investigated microbial interactions within plastic-associated biofilms across marine, freshwater and wastewater environments using 16S rRNA gene sequencing coupled with machine learning approaches. Their study examined the microbial diversity of plastisphere communities while exploring the ecological interactions between putative plastic-degrading bacteria (PDBs) and non-degrading bacteria (NDBs). The authors identified habitat-specific assemblages of potential plastic degraders and demonstrated that plastic degradation is likely driven by cooperative interactions between degrading and non-degrading microorganisms rather than by individual taxa alone. Wastewater plastisphere communities exhibited the highest microbial diversity, highlighting the strong influence of habitat on community assembly. Overall, the study emphasizes the ecological complexity of plastisphere communities and the importance of adopting community-level approaches to understand plastic biodegradation, while demonstrating the potential of machine learning to identify key microbial interactions that may support the development of more efficient plastic-degrading microbial consortia.

Complementing this focus on bacterial biofilms, Guo et al. examined benthic diatom communities, key primary producers within river biofilms, along the middle and lower reaches of the Yellow River. Combining diversity metrics with structural equation modeling, the authors found that water quality, particularly nutrient concentrations and turbidity, was the main driver of diatom taxonomic composition and diversity, with geography and land use acting mostly indirectly through their influence on water quality. The middle reaches, with moderate nutrient inputs and more stable hydrology, supported higher diversity and taxa typical of meso-/oligotrophic conditions, whereas the more urbanized and nutrient-enriched lower reaches were dominated by eutrophic-tolerant genera and greater community homogenization. These findings illustrate how anthropogenic pressures reshape the primary-producer component of river biofilms and offer a transferable framework for biofilm-based bioindicators in other large, human-impacted rivers.

In the paper by Alouadi et al., the authors investigated the poorly characterized microbial diversity and functional potential of marine biofilms through the study of culturable epibiotic bacteria associated with biofouling organisms. The authors combined DNA barcoding of fouling organisms with the characterization of biochemical and antimicrobial activities of bacterial isolates from an underexplored region of the southern Mediterranean. This approach displayed a high taxonomic diversity among the studied culturable epibiotic bacteria, including underexplored taxa in marine epibiotic studies which may reflect a broad diversity of lifestyles among these microorganisms. Results also showed a high frequency of hydrolytic enzyme production, such as DNases, lipases and proteases, that play a role in organic-matter degradation and biofilm remodeling and may be underreported in marine biofilms. Furthermore, the authors detected antibiotic-resistant strains alongside antimicrobial-producing strains. This antibiotic resistance was particularly prevalent among isolates belonging to clinically relevant genera, further emphasizing possible health risks of aquatic biofilms as reservoirs of antibiotic resistance.

Stehlik et al. studied the effect of environmental factors on microbial growth and biofilm formation, applying nonlinear and mixed-effects modeling approaches that remain underused in aquatic microbiology. Specifically, the authors used nonlinear, GAMLSS, and mixed-effects models to characterize a broader range of microbial growth responses. For this purpose, a strain of the bacterium *Cobetia marina* was selected for its capacity for both planktonic and biofilm growth on surfaces. Results demonstrated that pH had a marked impact on *Cobetia marina* dynamics, resulting in higher biofilm formation at higher pH but lower planktonic growth. This suggests that ocean acidification could be an important factor shaping microbial population and biofilm dynamics and adaptation to changing environmental conditions. At the same time, the authors showed that temperature differently regulates planktonic growth and biofilm formation of *C*. *marina*. Specifically, above the optimal growth temperature, both microbial growth and biofilm formation decline, while below the optimum, microbial growth decreases as biofilm formation increases. As a methodological conclusion, the authors suggest that these nonlinear models could help predict growth and biofilm formation under field conditions for a range of microorganisms. Therefore, this approach may be useful for forecasting biofilm dynamics in marine environments, particularly under climate change scenarios.

The work of Tejero et al. establishes a simple and reproducible *in vitro* platform for studying biofilm formation by *Tenacibaculum maritimum*, one of the most important bacterial pathogens affecting marine aquaculture, which causes tenacibaculosis. Recognizing that biofilm formation by this bacterium contributes to pathogen persistence and antimicrobial tolerance, the authors optimized experimental conditions for consistent biofilm development using polystyrene microplates. The method provides an accessible assay for evaluating antibiofilm compounds and addresses the limited availability of current methods for analyzing *T*. *maritimum* biofilm growth. The results showed that gilthead seabream (*Sparus aurata*) skin mucus exhibits a marked concentration-dependent inhibitory effect on both planktonic growth and biofilm formation, while displaying a more limited capacity to disrupt mature biofilms. These findings reinforce the protective role of fish skin mucus and provide a valuable experimental framework for investigating host-derived antibiofilm factors against *T. maritimum*. These findings support the importance of the development of control strategies for biofilm-related diseases in aquaculture.

Collectively, the studies presented in this Research Topic advance our understanding of the diversity, ecology, dynamics, and functions of aquatic biofilms across natural and engineered environments. By providing new insights into biofilm formation, microbial interactions, environmental drivers, antimicrobial resistance, and strategies for biofilm prevention and control, these insights contribute to the development of more effective approaches for managing drinking water systems, mitigating biofouling and microbiologically influenced corrosion in engineered infrastructures, and protecting aquatic ecosystem and public health (Figure 1).
